# Local Use of Bromide of Potassium*Read before the Louisville Academy of Medicine, December 17, 1875.

**Published:** 1876-08

**Authors:** Martin F. Coomes

**Affiliations:** Assistant to the Chair of Ophthalmology and Otology in the Hospital College of Medicine


### LOCAL USE OF BROMIDE OF POTASSIUM.*

BY MARTIN F. COOMES, M.D.,

Assistant to the Chair of Ophthalmology and Otology in the Hospital College of Medicine.

The bromide of potassium, in substance or saturated solution, applied to living muscular tissue produces paralysis. The same effects are produced when it is applied to a nerve trunk, or injected into an artery; that is, the muscles supplied by the nerve or artery which the drug has acted in or upon will be paralyzed.†

Applied to mucous surfaces, it is a local anæsthetic, although this effect is secondary unless used sn weak solution, say ten or fifteen grains to the ounce of water. The action of the bromide, when applied to mucous surfaces, in substance or saturated

*Read before the Louisville Academy of Medicine, December 17, 1875.

†See Stille's Work on Therapeutics.

solution, resembles that of a caustic. Its effects upon mucous surfaces are not visible like those of an ordinary caustic. It does not whiten the tissues, nor is its application painless, as is the case with many caustics. When applied to the schneiderian membrane, or palpebral conjunctiva, the pain is severe and of a hot burning character. The larynx and fauces are more tolerant to its action than the eye or nose, but the pain is similar in being associated with heat. The duration of the pain is never more than a few seconds. Applied to congested mucous surfaces, it disgorges the distended vessels, and increases the secretive action of the mucous follicles.

In papillary ophthalmia, commonly called "granular lids," the results of its action are similar to those obtained from the use of the muraite of ammonia. It reduces the hypertrophy, increases the amount of secretion, and allays pain. Its anæsthetic properties alone give it an advantage over the ammonia.

In the treatment of nasal catarrh, where there is a dry condition of the membrane, the bromide, in powder or saturated solution, is an agent of great value. Where there is hypertrophy of the membrane lining the nasal cavities, with an insufficient amount of the normal secretions, a condition met with in poliferous inflammations of the membrane, insufflations of the powdered bromide or injections of the saturated solution produce excellent results. By its use the secretions of the membrane are increased, congestion lessened, and a marked reduction in the hypertrophied tissues. Its immediate effects in these cases of poliferous inflammation of the nasal cavities is to relieve the patient of that sense of "*stuffiness*" which is most always complained of.

For the last year and a half I have relied almost entirely upon the bromide of potassium as a local agent in the treatment of throat affections. It has but rarely disappointed me. The results which I have obtained from its use in this class of diseases have been most gratifying. In cases of acute tonsilitis and pharyngitis, it matters not whether in their incipiency or in the advanced stages, a solution of the bromide of potassium, sixty grains to the ounce of water, applied with a mop or with an atomizer every hour or two, will be found to produce well-nigh complete relief. In cases of ulceration the open sore should be touched with carbolic acid or nitrate of silver. In but few cases will it be necessary to re-apply the escharotic a second time. Under this plan of treatment all the painful and distressing symptoms that attend such cases speedily disappear.

In every instance the patients treated with the bromide expressed themselves as feeling great relief immediately after the application of the drug. These statements have been verified by the rapid reduction of temperature in the affected part, the restoration of the functions in the mucous follicles in the vicinity, the disgorgement of the distended blood-vessels, and almost an entire absence of pain during the whole course of the disease.

In affections of the larnyx it is equally applicable. I have seen in the larnyx a polypus of considerable magnitude disappear by the application of the powdered bromide of potassium once a day at first, then every third day for a month or six weeks. The tumor was large enough to fall across the vocal cords and produce almost complete aphonia. After the polypus disappeared the patient was subject to attacks of dysphonia upon the least exposure to damp weather. The voice in this case was always partially and temporarily restored by the use of the bromide, but it seemed to exercise no permanent curative effect.

In cases of congestion of the laryngeal mucous membrane, attended with cough, the application of a forty or sixty-grain solution of the bromide with the atomizer, is an excellent remedy.

I have used it in the cough of phthisis with encouraging results. I have not been able to give it a fair trial in this class of diseases on account of the number of my cases being too small to draw any definite conclusions. In the advanced stages of phthisis, when the cough is severe, and the amount of irritability about the epiglottis is so excessive as to excite retching and vomiting, I think it is preferable to cough-mixtures.

My experience with the bromide in cases of œdema of the glottis has been very limited, having only seen one case, and that in twenty hours after the first application of the drug. The patient was under the care of my friend, Dr. D. S. Reynolds. He told me that when he first saw the patient her face was livid, countenance anxious, and a state of general excitement prevailed. The glottis was almost closed, the chink not being larger than a quarter of an inch in diameter. To produce this state of things it is left for the reader to imagine the amount of effusion which must necessarily take place to bring it about. The doctor applied the powdered bromide directly to the œdematous parts with a brush, with the effect of relieving his patient in the short space of twenty minutes. I saw the patient on the following evening, and to all appearances she was well, her breathing perfectly easy, and the countenance presenting its natural appearance, whereas but a few hours before she appeared to be suffocating, with eyes aglare and face of purple hue.

Its application might be somewhat difficult in children. In cases where the drug cannot be used in powder with a brush, the tongue may be drawn forward, and as much as a teaspoonful of the saturated solution poured on its base, and the organ held until the patient begins to strangle, which will be as soon as the solution reaches the larnyx. This seemingly cruel mode of administration may appear to be attended with danger, but there can be nothing more than temporary strangulation, which will excite violent coughing, an act that may in all probability cause the distended membrane to rupture, and in this way relieve the patient. Free incision of the œdematous membrane will afford relief; but it requires an experienced hand to make the incision. In spasmodic affections of the glottis, I think the bromide promises to be an invaluable agent. I have used it in one case of spasmodic croup, with the happiest result. The patient was a very fleshy child, six years old, and was subject to such attacks. When I first saw her she had the croupy cough, with difficult breathing and livid face. I sprayed her throat with a solution of the bromide of potassium, sixty grains to the ounce of water. She was relieved of all the unpleasant symptoms at once. I ordered the application to be repeated if the symptoms returned. The repetition was not called for until seven hours subsequently, when upon a return of the spasmodic respiration the spray was repeated, and the disease did not again return.—*Louisville Med. News.*

